# Rapid SARS-CoV-2 testing in primary material based on a novel multiplex RT-LAMP assay

**DOI:** 10.1371/journal.pone.0238612

**Published:** 2020-11-02

**Authors:** Bernhard Schermer, Francesca Fabretti, Maximilian Damagnez, Veronica Di Cristanziano, Eva Heger, Sita Arjune, Nathan A. Tanner, Thomas Imhof, Manuel Koch, Alim Ladha, Julia Joung, Jonathan S. Gootenberg, Omar O. Abudayyeh, Volker Burst, Feng Zhang, Florian Klein, Thomas Benzing, Roman-Ulrich Müller

**Affiliations:** 1 Department II of Internal Medicine, University of Cologne, Faculty of Medicine and University Hospital Cologne, Cologne, Germany; 2 Center for Molecular Medicine Cologne, University of Cologne, Faculty of Medicine and University Hospital Cologne, Cologne, Germany; 3 Cologne Excellence Cluster on Cellular Stress Responses in Aging-Associated Diseases (CECAD), University of Cologne, Cologne, Germany; 4 Institute of Virology, Faculty of Medicine and University Hospital Cologne, University of Cologne, Cologne, Germany; 5 New England Biolabs, Ipswich, MA, United States of America; 6 Institute for Dental Research and Oral Musculoskeletal Biology, Center for Biochemistry, University of Cologne, Cologne, Germany; 7 Howard Hughes Medical Institute, Cambridge, MA, United States of America; 8 Broad Institute of Massachusetts Institute of Technology and Harvard, Cambridge, MA, United States of America; 9 McGovern Institute for Brain Research at Massachusetts Institute of Technology, Cambridge, MA, United States of America; 10 Department of Biological Engineering, Massachusetts Institute of Technology, Cambridge, MA, United States of America; 11 Massachusetts Consortium for Pathogen Readiness, Boston, MA, United States of America; 12 Emergency Department, Faculty of Medicine and University Hospital Cologne, Cologne, Germany; 13 Department of Brain and Cognitive Sciences, Massachusetts Institute of Technology, Cambridge, MA, United States of America; 14 German Center for Infection Research, Partner Site Bonn-Cologne, Cologne, Germany; University of Helsinki, FINLAND

## Abstract

**Background:**

Rapid and extensive testing of large parts of the population and specific subgroups is crucial for proper management of severe acute respiratory syndrome coronavirus 2 (SARS-CoV-2) infections and decision-making in times of a pandemic outbreak. However, point-of-care (POC) testing in places such as emergency units, outpatient clinics, airport security points or the entrance of any public building is a major challenge. The need for thermal cycling and nucleic acid isolation hampers the use of standard PCR-based methods for this purpose.

**Methods:**

To avoid these obstacles, we tested PCR-independent methods for the detection of SARS-CoV-2 RNA from primary material (nasopharyngeal swabs) including reverse transcription loop-mediated isothermal amplification (RT-LAMP) and specific high-sensitivity enzymatic reporter unlocking (SHERLOCK).

**Results:**

Whilst specificity of standard RT-LAMP assays appears to be satisfactory, sensitivity does not reach the current gold-standard quantitative real-time polymerase chain reaction (qPCR) assays yet. We describe a novel multiplexed RT-LAMP approach and validate its sensitivity on primary samples. This approach allows for fast and reliable identification of infected individuals. Primer optimization and multiplexing helps to increase sensitivity significantly. In addition, we directly compare and combine our novel RT-LAMP assays with SHERLOCK.

**Conclusion:**

In summary, this approach reveals one-step multiplexed RT-LAMP assays as a prime-option for the development of easy and cheap POC test kits.

## Introduction

The recent pandemic of SARS-CoV-2 is a major challenge for healthcare systems worldwide. Lacking an effective and approved vaccine, reliable screening of samples from nasopharyngeal swabs for viral RNA is a fundamental pillar for effective disease control. Governmental measures (e.g. shutdown of social life) largely depend on such data to allow for a rapid adaptation to changes in epidemiologic indicators. Furthermore, isolation and quarantine of infected and exposed individuals are key for effective outbreak control. Here, the prompt establishment of a diagnostic real-time PCR based testing [1] has been instrumental already in the early phases of the pandemic. Such qPCR-based tests are extremely powerful due to their high sensitivity and specificity. However, these assays require specific lab equipment and expertise which is typically not widely available directly at the point-of-care making transportation to a specialized facility necessary. Furthermore, the material required for the different steps involved may become subject to shortages during a pandemic making alternative approaches an important goal. Even though RNA isolation and subsequent qPCR is performed in only a few hours, actual turnaround times from sample collection to diagnostic test results are often much longer. Decision-making in both healthcare facilities and other spheres of public life would be facilitated enormously by direct testing on site with short turn-around times. Such an approach could also limit the need for quarantine, allow healthcare workers after exposure to continue their work upon a daily negative swab and avoid shortages in systemically relevant personnel. Recently, a number of PCR-independent methods have been proposed for this purpose including isothermal amplification (RPA, LAMP [2–12]) and their combination with genome editing tools such as Cas12a [13] or Cas13a [14, 15] for improved performance. The majority of these studies, however, have not been carried out on direct primary material but on RNA isolated from patient samples or generated in vitro [3–9, 13]. In the study at hand, we use previously described RT-LAMP and SHERLOCK assays on both isolated RNA and primary material from patients [9, 14]. In addition, we describe a newly designed multiplexed RT-LAMP assay targeting Orf3a and Orf7a of SARS-CoV-2. Orf3a and Orf7a have been selected since these targets have not been used for any available diagnostic qPCR assay, to avoid the risk of amplicon cross-contamination between different types of assays.

## Methods

### Samples in universal transfer medium and isolated RNA

Nasopharyngeal swabs were taken from symptomatic patients presenting at University Hospital of Cologne from March to April 2020. Swabs were directly transferred in 1–3 ml of universal transfer medium (UTM) or PBS. For diagnostic qPCR, RNA was extracted from 500 μl UTM / PBS of the swab samples using the automated MagNA Pure 96 system (Roche). Diagnostic qPCR was performed using a RealStar SARS-CoV-2 RT-PCR kit 1.0, with primers targeting E and S gene (Altona Diagnostics) on a LightCycler 480 (Roche). Positively and negatively tested UTM/PBS samples were randomly selected for RT-LAMP or SHERLOCK assays. Here, 0.5 to 1.9 μl isolated RNA or 0.5 to 1.9 μl UTM/PBS was used for subsequent RT-LAMP/Sherlock assays. This study was performed exclusively with surplus diagnostic material that was analyzed anonymously. No specific approval number from the IRB (Ethikkommission der Medizinischen Fakultaet der Universitaet zu Koeln (IRB of University of Cologne)) was required.

### Sample inactivation for RT-LAMP and Sherlock

For assays from direct material 10 μl of UTM/PBS were incubated at 98°C for 15 minutes in a PCR cycler with heated lid placed inside a safety cabinet. Treatment of 15 min at 92°C has been shown to inactivate SARS-CoV-2 [16] and 98°C was found to be preferable for downstream RNA-based applications.

### LAMP primer design

For Orf1a and Gene N we used previously described primer sets [9]. Additional primer sets were designed for Orf3a, Orf7a and the M gene using the primerexplorer V5 tool (http://primerexplorer.jp/e/). All primers were ordered from IDT, purified with standard desalting, as PAGE purification did not improve the performance of the assays. All primer sequences are listed in S1 Table.

### RT-LAMP assay

All RT-LAMP reactions were assembled on ice. In brief, each 20 μl reaction contained 10 μl WarmStart Colorimetric RT-LAMP mix (NEB), 2 μl primer mix (F3/B3 2 μM each; FIP/BIP 16 μM each; LF/LB 4 μM each), 40 mM guanidine hydrochloride (from a 4 M stock solution, pH8), DNase/RNase free water and 0,5 μl sample. For multiplexed RT-LAMP the additional primer mix replaced 2 μl of DNase/RNAse free water. Initially, the assays were performed without guanidine hydrochloride at 65°C for 40 minutes, while the multiplexed reaction was done at 60°C for 40 to 50 minutes, as indicated in the figures. Each assay was performed including several negative controls. Positive reactions were identified due to a clear change in color from pink/red to orange/yellow. In two cases out of almost 200, the mere addition of 0.5 μl sample to the reaction resulted in a color change. These samples are not included in the data shown.

### SHERLOCK assay with RPA

The two step SHERLOCK assay for Orf1a and S -gene was performed according to the protocol established in Feng Zhang’s lab at MIT (https://zlab.bio/s/COVID-19-detection-v20200321.pdf) [14, 15]. In brief, an RPA reaction was set up using 5.9 μl RPA mix (Twist AMP), 0.2 μl Protoscript RT (NEB), 1μl RPA primer mix (10 μM each), 1.9 μl sample (isolated RNA or UTM/PBS) and 0.5 μl magnesium acetate (280 mM stock solution). The reaction was mixed, spun down and incubated 42°C for 25 min. The Cas13a-based detection step was performed in a 20 μl reaction as follows: 2 μl TRIS-HCl buffer (400 mM, pH 7.4), 9.6 μl DNase/RNase free water, 2 μl LwaCas13a (corresponding to 120 ng of protein), 1 μl crRNA (10 ng/μl), 1 μl Lateral-Flow-Reporter (20 μM), 1 μl SUPERase In RNase Inhibitor (Thermofisher Scientific), 0.6 μl T7 Polymerase (Lucigen), 0.8 μl rNTPs (25 μM each; NEB), 1 μl MgCl2 (120 mM stock solution) and 1 μl of previous RPA reaction. The reaction was mixed and spun down, then incubated at 37°C for 30 min. Next, the reaction was diluted with 80 μl of HybriDetect Buffer (Milenia), mixed, and then a HybriDetect dipstick (Milenia) was placed in the reaction tube and incubated at RT. Results were visible within 5 minutes.

### Cas13a combined with RT-LAMP

To combine the RT-LAMP amplification with Cas13a detection we added a T7 promoter in the loop region of the FIP primers for the RT-LAMP assay (S1 Table). After the RT-LAMP reaction at 65°C for 40 min, 0.5 μl were used in the Cas13a detection assay assembled as described above, using a specific crRNA targeting the RT-LAMP Gene N amplicon.

## Results

To allow for the comparison of different nucleic acid detection methods for SARS-CoV-2 we collected redundant material from nasopharyngeal swabs obtained for qPCR testing in clinical routine due to suspected COVID-19. 171 samples were selected randomly from samples collected in the period from March to April 2020. The cohort included individuals between the age of 1 month and 88 years with a close to equal distribution of men and women (S2 Table).

### SHERLOCK and RT-LAMP on isolated RNAs

We first tested two recently described assays for SARS-CoV-2 detection on isolated RNA from patient samples. Specifically, we performed colorimetric RT-LAMP assays using primers targeting Orf1a and Gene -N [9] and two-step SHERLOCK assays combined with lateral flow detection [14, 15] targeting Orf1a and S gene (S1A–S1C Fig). Even though both assays worked well on these samples, they failed to detect the virus in specimen that were positive in diagnostic qPCR at cycle threshold (Ct) values > 30 for E and S gene (S1 Fig). Moreover, RT-LAMP appeared to be slightly more sensitive and specific (S1C Fig).

### RT-LAMP assays on primary material from nasopharyngeal swabs

**Fig 1:**
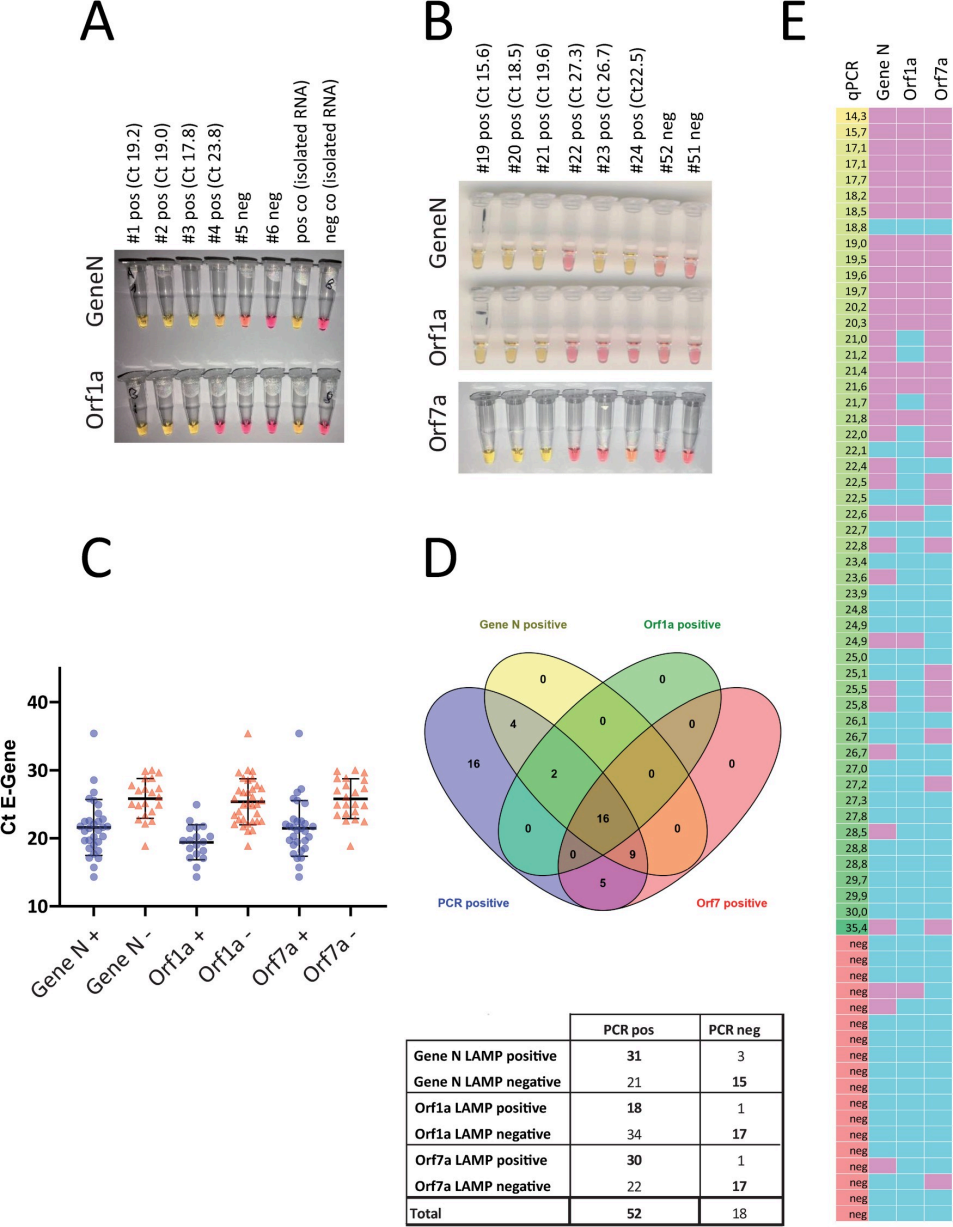
Detection of viral RNA with RT-LAMP. A. Representative pictures of a RT-LAMP assay. UTM samples from 6 patients, 4 of which tested positively for SARS-CoV-2 in diagnostic qPCR were analyzed by RT-LAMP targeting Gene N and Orf1a. Isolated RNA from swabs served as positive and negative control. B. 70 samples were analyzed with three different RT-LAMP assays targeting Gene N, Orf1a and Orf7a. Representative results from additional assays. C. The individual value plot shows of Ct values of the diagnostic qPCR for the E gene of positive and negative RT-LAMP assays. D. Total number of positive and negative RT-LAMP assays from patient samples tested positive or negative for SARS-CoV-2. E. Heat map summarizing the results of three different RT-LAMP assays on all 70 samples (left column: Ct value for E gene from diagnostic PCR in ascending order; other columns: pink indicating positive, blue indicating negative result. Grey: not performed).

Due to limited availability of the RPA reagents required for the first step of SHERLOCK at that time paired with the results described above, we focused our efforts on validation of the RT-LAMP assays. Since detection worked on isolated RNA, we went on to test this approach using primary material (i.e. transport medium from nasopharyngeal swabs without RNA isolation) Strikingly, both RT-LAMP assays performed well on these specimens. Using as little as 0.5 μl UTM as input for each reaction to avoid inhibitory effects of the medium or of tissue contaminants on the reaction, detection worked in samples tested positive by qPCR (see Ct as reference) (Fig 1A).

### Establishment of primer sets for novel target genes in RT-LAMP assays

In parallel, 5 additional sets of RT-LAMP primers targeting additional genes of the SARS-CoV-2 genome (NC_045512) were designed, tested and validated. Here, we preferred genes that are not target of any standard diagnostic qPCR assay, to minimize and avoid any possible interference of such point of care assays with other routine diagnostic pipelines. Among these new primer sets, the oligos targeting Orf7a showed the highest sensitivity and specificity in several tests on diluted isolated RNA and was thus selected for further testing in primary material. In this second set of experiments we screened a total of 70 samples, 52 of which had been tested positive for SARS-CoV-2 by diagnostic qPCR. Fig 1B displays representative results of the RT-LAMP assays targeting Gene N, Orf1a and Orf7a. Assays targeting Gene N and Orf7a were more sensitive than the one targeting Orf1a as indicated by the mean Ct value of RT-LAMP-positive samples (Fig 1C), by the total number of samples that were correctly identified (Fig 1D and 1E). 3 out of 70 specimens turned out positive for Gene N and 1 out of 70 for Orf1a and Orf7 respectively, that were negative by qPCR.

### Increasing the sensitivity of RT-LAMP

In order to increase the sensitivity of RT-LAMP assays to a comparable level with qPCR we performed a “two-step” LAMP reaction, either using a small amount (0.2 to 0.5 μl) of a first RT-LAMP reaction as template for a second one or by refreshing the first reaction with new enzymes and dNTPs. All of these attempts resulted consistently in negative controls (water, empty UTM or samples from negative patients) turning positive, either because of unspecific amplification or because of the necessary re-opening of the reaction tubes after the first amplification step. The use of mineral oil on top of every reaction–performed to avoid cross-contamination–did not improve these results. In an additional set of experiments, we combined the RT-LAMP assays targeting Gene N with LwaCas13a mediated detection. To this end, we added a T7 promoter in the loop region of the FIP-primer and designed a crRNA targeting the amplicon of the RT-LAMP reaction. The addition of the T7 promotor did not affect the efficiency of the RT-LAMP reaction (S2A Fig) and a positive LwaCas13a mediated detection is indicated by the upper band in the lateral flow assay (S2B Fig). While this combination of RT-LAMP and LwaCas13a provides an additional proof of specificity as compared to the RT-LAMP reaction alone, sensitivity was not increased. An additional possibility to improve sensitivity of RT-LAMP is multiplexing, using primer mixes for different genes of the viral genome in the same reaction. After testing different combinations of these primer sets, we observed superior performance of RT-LAMP assays containing primer sets amplifying Orf7a and Orf3a (primer set A) combined with a lower reaction temperature (S3 Fig).

### Multiplex RT-LAMP assay on clinical samples

**Fig 2:**
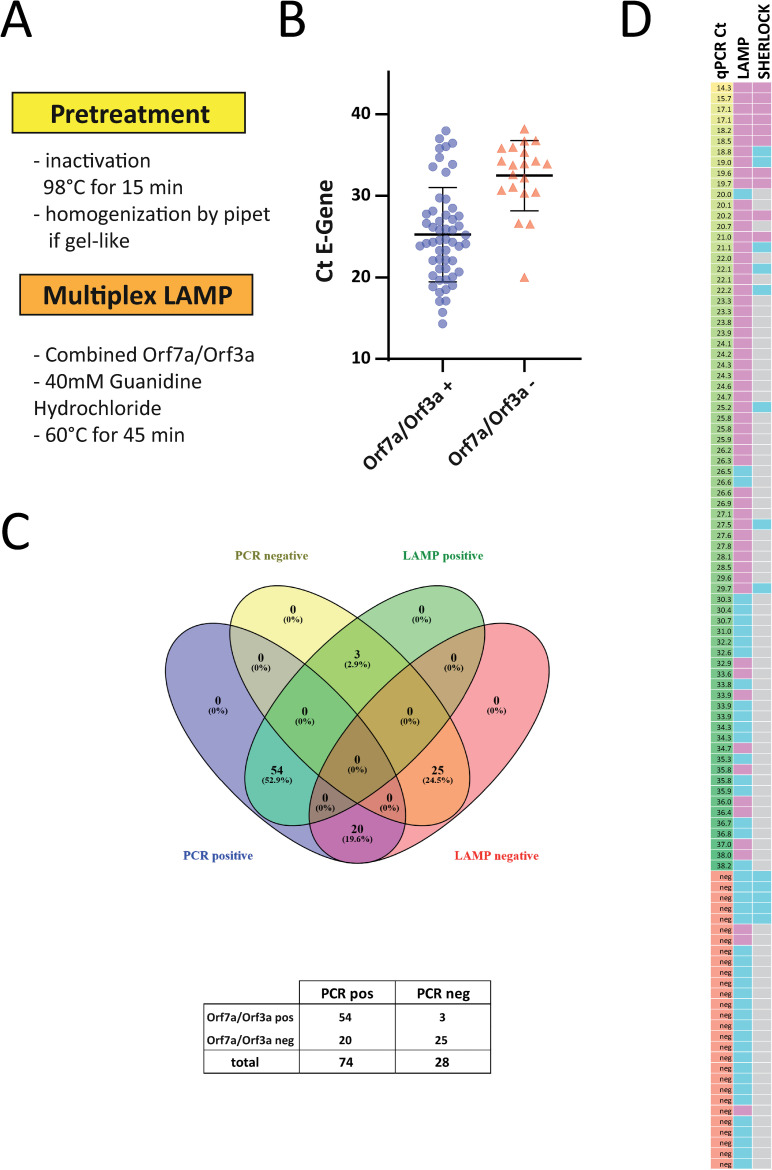
Detection of viral RNA with multiplexed RT-LAMP. A. Workflow of the optimized multiplexed RT-LAMP protocol. B. 104 samples were analyzed with the multiplexed RT-LAMP assay targeting Orf7a and Orf3a. The individual value plot shows Ct values of the diagnostic qPCR for the E gene of positive and negative RT-LAMP assays. C. Total number of positive and negative multiplexed RT-LAMP assays from patient samples tested positive or negative for SARS-CoV-2. D. Heat map summarizing the results (left column: Ct value for E gene from diagnostic PCR in ascending order; middle and right column: pink indicating positive, blue indicating negative result. Grey: not performed).

To increase accessibility of RNA and efficiently inactivate the virus we incubated UTM from swabs at 98°C for 15 min. Treatment at 92°C for 15 minutes has been demonstrated to efficiently inactivate SARS-CoV-2 [16], while 98°C appeared a good choice for downstream RNA applications in case of SARS-CoV-2 [17]. After incubation at 98°C, some of the UTM samples showed a gel-like consistency. This was observed in only a minor fraction of the samples and was most likely caused by protein and sugar supplements contained in one specific type of UTM. This UTM was easily recognizable since it was the only one containing phenol red as pH indicator. In these cases, pipetting about 10 times up and down with a P20 pipette allowed for complete homogenization and subsequently accurate pipetting. Again, we only used 0.5 μl UTM for each reaction. In addition, we added guanidine hydrochloride as a classical RNAse inhibitor and LAMP enhancer [18] to the multiplexed reaction at 60°C. These modifications in sample preparation combined with our new multiplexed assay were used to analyze a set of 102 clinical samples consisting of 74 SARS-CoV-2 positive and 28 negative swabs (Fig 2). 54 SARS-CoV-2 positive samples with Ct values up to 38 were positive in the RT-LAMP assay (Fig 2B), while still 20 out of 74 qPCR positive samples were not detected in our assay on direct material. However, the vast majority of samples up to a Ct of 30 were correctly identified (94%; 45 out of 48). Meanwhile, RPA reagents arrived and we performed the two step SHERLOCK assays on some of the very same direct samples. However, sensitivity was much lower with a cut-off threshold of 21 cycles (Fig 2D; representative assays shown in S4 Fig). In summary, our multiplex RT-LAMP protocol is a simple and sensitive way to detect SARS-CoV-2 RNA from clinical samples.

## Discussion

Based on our data we conclude, that multiplexing primers in RT-LAMP reactions is a highly promising way to further increase sensitivity of these assays and to quickly develop a rapid POC test. Currently, a test based on our multiplexed RT-LAMP assay would–in contrast to a good specificity—most likely miss to identify those infected patients with very low amounts of viral RNA in the nose or throat and would not yet reach the sensitivity of the gold-standard qPCR assays. However, the RT-LAMP approach comes with several clear-cut advantages. In contrast to classical qPCR, it can be used in primary material without the need for RNA isolation and yields results rapidly. Furthermore, only little equipment is required (thermoblock) and could easily be made available in e.g. emergency rooms. The estimated costs for reagents are below 1.50 € per reaction with the advantage–compared to qPCR–that additional expenses for nucleic acid isolation are not required. Regarding the target setting of primary screening, one could hypothesize, that identifying patients with high viral loads would detect those individuals that are highly infectious. Consequently, in this setting, even a POC test with a lower sensitivity as compared to diagnostic qPCR would be extremely useful and could be backed up by a combination with qPCR the results of which get available later. Currently, a relation between the level of viral RNA in swabs and the infectivity of a patient has not yet been definitively established. Detection of viral RNA is not equivalent to detection of infectious virus. One retrospective study, however, suggested low infectivity of patients with Ct-values >24 in E gene qPCR from swabs since the authors did not observe viral growth in exposed Vero cells [19]. Besides, a statement paper of the National Centre for Infectious Diseases and the Chapter of Infectious Disease Physicians (Singapore) refers to a study with 73 COVID-19 patients where a Ct >30 was found to be the threshold of infectivity [20]. Additional studies report Ct values between 31 and 34 as cut-off for infectivity in different set-up [21, 22]. A recently updated meta-analysis provides a very comprehensive overview on this important topic supporting the conclusion that infectivity is related to the cycle threshold level, but a clear cut-off can currently not yet be defined [23]. Nonetheless, the ultimate goal would be a POC test that reaches the sensitivity of qPCR and can completely replace the current approach where favorable. Studies in the near future will clarify, whether patients with high viral RNA loads are indeed the most contagious individuals. Besides, this knowledge will also be of greatest importance for the actual clinical interpretation of qPCR results in the future. By now, it has been shown that the viral load is already high before and maybe highest at onset of symptoms [24] and at the time point of presentation to the clinic followed by a steady decline [25]. Consequently, when focusing the screening on pre-symptomatic individuals, sensitivity of the RT-LAMP assay may actually be higher than in the current cohort. Additionally, one thing to be kept in mind when directly comparing sensitivity between the different methods, is the fact that qPCR reaches this standard only in isolated RNA whilst the multiplex RT-LAMP assay attains an optimized detection rate already in primary material. Of note, further addition of a step concentrating RNA using bead-based pulldown to an RT-LAMP-based protocol has also been successful [26]. Remarkably, here the authors move from swabs, that require trained personnel and personal protection equipment, to a home-based gargle [26] and a very recent report using qPCR demonstrated, that even saliva could be used as a valid source for viral diagnostics [27]. Optimizing also the collection of samples and including such specimens will be an important step towards broadly applicable POC testing. Regarding specificity, only few specimens turned out to be positive in the multiplex RT-LAMP assay that were negative in qPCR. Since qPCR is the gold-standard this can be interpreted as a minor limitation in specificity. However, qPCR itself does not reach a sensitivity of 100% [28]. Consequently, it is not clear yet whether these individuals were truly negative or missed by the qPCR assay.

CRISPR/ Cas12 [13] or Cas13a [15] based assays are another promising way to detect RNA in a PCR-independent manner. Comparison of this approach with RT-LAMP demonstrates a surprisingly high sensitivity even of the colorimetric one step RT-LAMP assay. Very recently, a novel protocol for Cas13a - called STOP (‘SHERLOCK Testing in One Pot’)—has been described [29]. As this replaces the isothermal RPA reaction of the original SHERLOCK protocol by a RT-LAMP reaction, the authors use a thermo-stable Cas13a enzyme to enable performing the entire reaction at the same temperature. Whilst being a very interesting approach, nonetheless, this comes with the difficulty the reaction tube has to be opened for the final lateral flow assay used for detection. In the real-world POC testing setting, this would require the establishment of a ‘pre-amp’ and ‘post-amp’ area to avoid cross-contamination, which may limit its use. Alternatively, lateral flow could be replaced by using a fluorescent probe together with an appropriate simple detection device. However, the highest Ct value resulting in a positive STOP assay is—at about 30 cycles—in a similar range compared to a recently described Cas12a-based method (DETECTR) [29]. The RT-LAMP assay described in our study works without re-opening the test tube after amplification and provides detection at higher Ct values. On the other side, both SHERLOCK and DETECTR add an additional level of specificity to the detection due to the crRNA directing the Cas12/13 enzyme. Both SHERLOCK and DETECTR require a considerable number of pipetting steps. In contrast, the multiplexed RT-LAMP assay demonstrates a similar or even higher sensitivity and requires only two simple pipetting steps at the POC: (1) taking a aliquot of the UTM for ‘boiling’ and (2) the addition of the 0.5 μl sample to the reaction tube, which could also be reached without a pipet by an inoculating loop. Reaction tubes can be prepared in anticipation elsewhere (e.g. in any nearby central facility) and stored for hours at 4°C. The preparation of these reaction tubes, is also done with only four simple pipetting steps (1. primer mix pre-diluted in water, 2. RT-LAMP mix, 3. guanidine hydrochloride, → aliquot in 200 μl tubes). This could even be further simplified by adding guanidine to the primer mix. After amplification, the tube and the according controls are photo-documented and discarded. In summary, we are convinced that systematically combining and testing different multiplex RT-LAMP primer sets on primary swab material is one of the most promising approaches to develop a powerful POC test. Transferring such assays to automated microfluidic formats [11] can become an important tool to support disease control strategies.

## Supporting information

S1 Fig Detection of viral RNA with RT-LAMP and SHERLOCK using isolated RNA from swabs. A. representative result from RT-LAMP assay targeting Gene N and Orf1a. Shift from red/pink to yellow/orange indicates a positive result (Ct value of E gene from diagnostic qPCR). B. Sherlock assay for S gene and Orf1a. The upper band indicates a positive result while the lower band is a control (Ct value of E gene from diagnostic qPCR). C. Summary of the RT-LAMP and Sherlock results on all available RNA samples (assay was regarded as positive with at least one positive result out of two RT-LAMP or SHERLOCK assays; left column: Ct value for E gene from diagnostic PCR in ascending order; green: positive result, blue negative result).
(TIF)

S2 Fig Detection of viral RNA with RT-LAMP and LwaCas13a. A. Results from the colorimetric RT-LAMP assay using 0,5 μl sample (UTM) with primers containing a T7 promotor in the loop region. B. 1 μl of the RT-LAMP reaction from A was entered in the Cas13a recognition reaction followed by lateral flow assay. Upper band indicates positive recognition of the Gene N target sequence.
(TIF)

S3 Fig Multiplexing of RT-LAMP reactions. Representative results from experiments trying to combine different RT-LAMP reactions in one tube. A multiplexed reaction targeting both Orf7a and Orf3a (set A) with a slight elongation of reaction time appears to be more sensitive and specific.
(TIF)

S4 Fig Direct comparison of multiplexed RT-LAMP with two-step SHERLOCK. A. Representative results from 8 samples analyzed by multiplexed RT-LAMP assay (Orf3a and Orf7a) and Sherlock (Orf1a and S gene). B. Summary of all RT-LAMP and Sherlock assays performed in parallel (SHERLOCK assay was regarded as positive with at least one positive result out of two assays; left column: Ct value for E gene from diagnostic PCR in ascending order; green: positive result, blue negative result).
(TIF)

S1 Table Sequences of all primers and RNAs used in this study. (PDF)

S2 Table Basic characteristics of the cohort. (PDF)
