# Supplementary figures and images for: Rapid SARS-CoV-2 testing in primary material based on a novel multiplex RT-LAMP assay
Source: PLoS One. 2020 Nov 2;15(11):e0238612. doi: 10.1371/journal.pone.0238612 (PMC7605681; doi:10.1371/journal.pone.0238612)

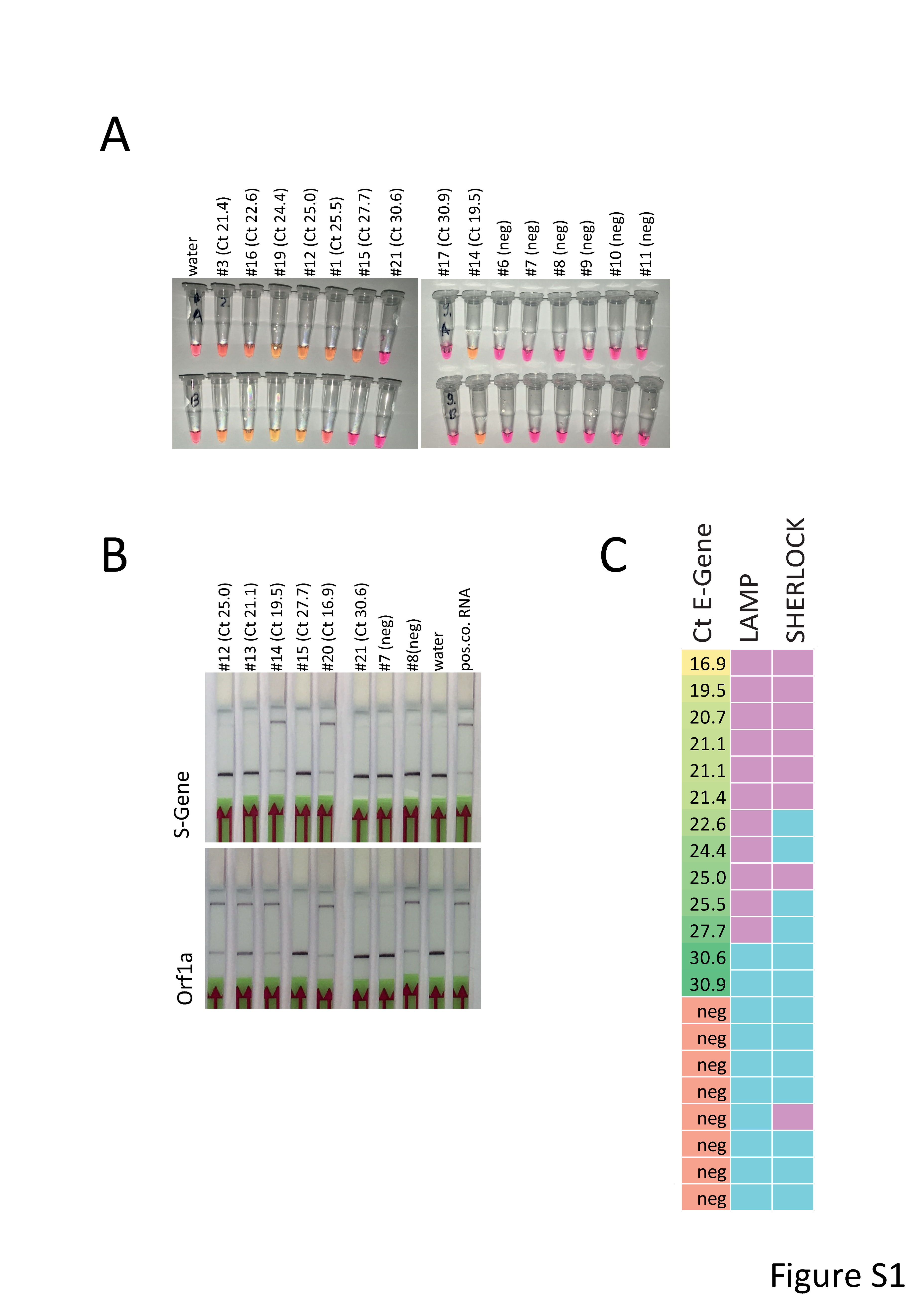

Supplement: S1 Fig — A. representative result from RT-LAMP assay targeting Gene N and Orf1a. Shift from red/pink to yellow/orange indicates a positive result (Ct value of E gene from diagnostic qPCR). B. Sherlock assay for S gene and Orf1a. The upper band indicates a positive result while the lower band is a control (Ct value of E gene from diagnostic qPCR). C. Summary of the RT-LAMP and Sherlock results on all available RNA samples (assay was regarded as positive with at least one positive result out of two RT-LAMP or SHERLOCK assays; left column: Ct value for E gene from diagnostic PCR in ascending order; green: positive result, blue negative result). (TIF) [file pone.0238612.s001.tif]

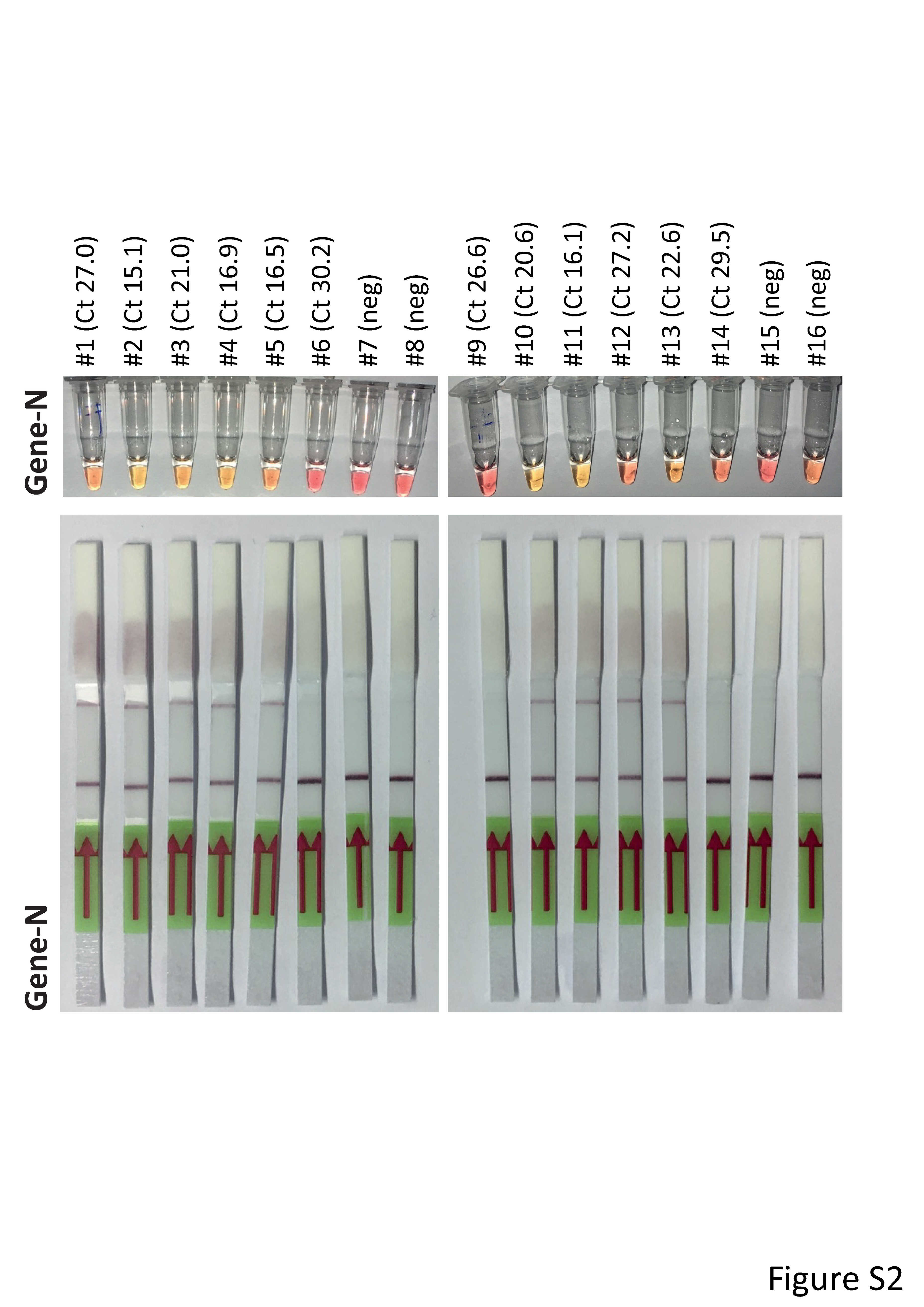

Supplement: S2 Fig — A. Results from the colorimetric RT-LAMP assay using 0,5 μl sample (UTM) with primers containing a T7 promotor in the loop region. B. 1 μl of the RT-LAMP reaction from A was entered in the Cas13a recognition reaction followed by lateral flow assay. Upper band indicates positive recognition of the Gene N target sequence. (TIF) [file pone.0238612.s002.tif]

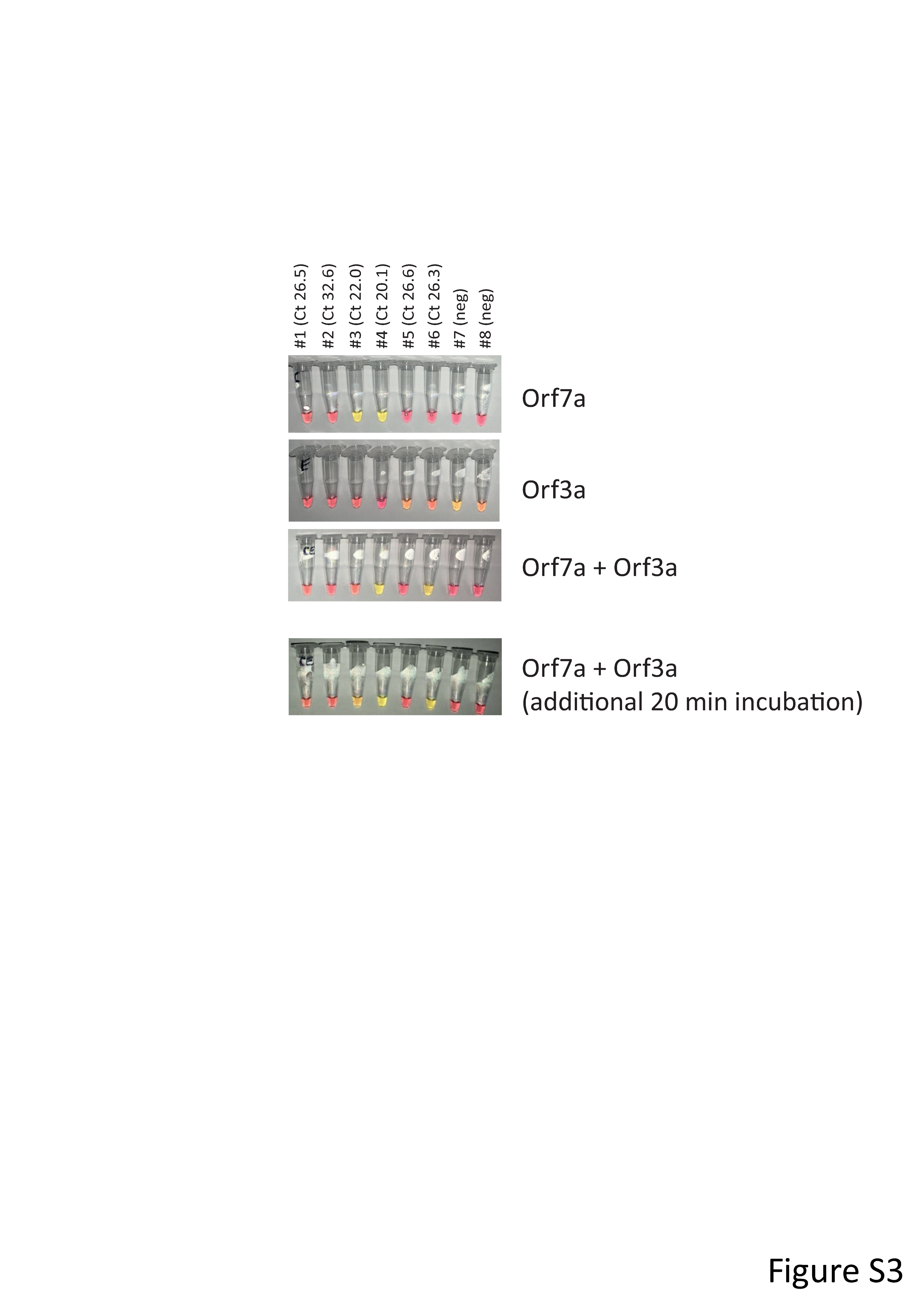

Supplement: S3 Fig — Representative results from experiments trying to combine different RT-LAMP reactions in one tube. A multiplexed reaction targeting both Orf7a and Orf3a (set A) with a slight elongation of reaction time appears to be more sensitive and specific. (TIF) [file pone.0238612.s003.tif]

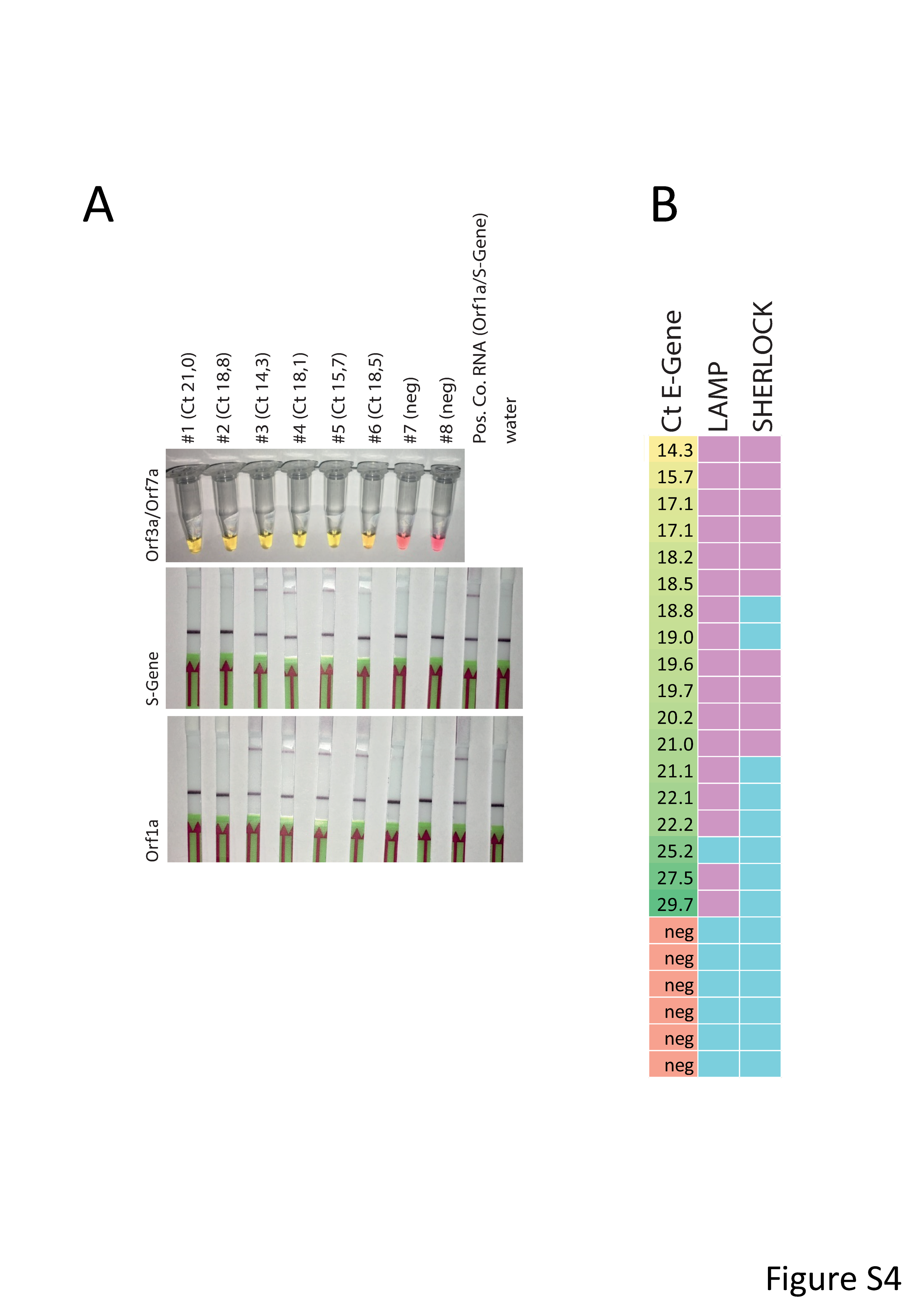

Supplement: S4 Fig — A. Representative results from 8 samples analyzed by multiplexed RT-LAMP assay (Orf3a and Orf7a) and Sherlock (Orf1a and S gene). B. Summary of all RT-LAMP and Sherlock assays performed in parallel (SHERLOCK assay was regarded as positive with at least one positive result out of two assays; left column: Ct value for E gene from diagnostic PCR in ascending order; green: positive result, blue negative result). (TIF) [file pone.0238612.s004.tif]
