# Supplementary material for: Rapid SARS-CoV-2 testing in primary material based on a novel multiplex RT-LAMP assay
Source: PLoS One. 2020 Nov 2;15(11):e0238612. doi: 10.1371/journal.pone.0238612 (PMC7605681; doi:10.1371/journal.pone.0238612)
Supplement: S1 Table — (PDF) [file pone.0238612.s005.pdf]

Primers		Sequence (5'-3')
**LAMP Orf1a[1]**	ORF1a-A-F3	CTGCACCTCATGGTCATGTT
	ORF1a-A-B3	AGCTCGTCGCCTAAGTCAA
	ORF1a-A-FIP	GAGGGACAAGGACACCAAGTGTATGGTTGAGCTGGTAGCAGA
	ORF1a-A-BIP	CCAGTGGCTTACCGCAAGGTTTTAGATCGGCGCCGTAAC
	ORF1a-A-LF	CCGTACTGAATGCCTTCGAGT
	ORF1a-A-LB	TTCGTAAGAACGGTAATAAAGGAGC
**LAMP GeneN[1]**	GeneN-A-F3	TGGCTACTACCGAAGAGCT
	GeneN-A-B3	TGCAGCATTGTTAGCAGGAT
	GeneN-A-FIP	TCTGGCCCAGTTCCTAGGTAGTCCAGACGAATTCGTGGTGG
	GeneN-A-BIP	AGACGGCATCATATGGGTTGCACGGGTGCCAATGTGATCT
	GeneN-A-LF	GGACTGAGATCTTTCATTTTACCGT
	GeneN-A-LB	ACTGAGGGAGCCTTGAATACA
**LAMP ORF7a**	ORF7a-F3	TCTTGGCACTGATAACACTC
	ORF7a-B3	GATCTGGCACGTAACTGAT
	ORF7a-FIP	TCCAGAAGAGCAAGGTTCTTTTAAA-GCTACTTGTGAGCTTTATCACT
	ORF7a-BIP	AGCTGATAACAAATTTGCACTGAC-GTTTTACGCCGTCAGGAC
	ORF7a-LF	GTTGTACCTCTAACACACTCTTGGT
	ORF7a-LB	GCTTTAGCACTCAATTTGCTTTTGC
**LAMP M GENE**	M-GENE-F3	AGTAATAGGTTTCCTATTCCTTAC
	M-GENE-B3	AGCCACATCAAGCCTACA
	M-GENE-FIP	GCCATAACAGCCAGAGGAAAATTAA-TGGATTTGTCTTCTACAATTTGC
	M-GENE-BIP	TTAGCTTGTTTTGTGCTTGCTG-ACAAGCCATTGCGATAGC
	M-GENE-LF	CAAAAACCTATTCCTGTTGGCATAG
	M-GENE-LB	CAGAATAAATTGGATCACCGGTGGA
	M-GENE-LB-2	GAATAAATTGGATCACCGGTGGA
**LAMP ORF3a-A**	ORF3a-A-F3	CACTTCAGACTATTACCAGCT
	ORF3a-A-B3	GCTTGTGCTTACAAAGGC
	ORF3a-A-FIP	GTTCTTCAGGCTCATCAACAATTTT-CTCAACTCAATTGAGTACAGACA
	ORF3a-A-BIP	CCAAATTCACACAATCGACGGTT-TAGTAGTCGTCGTCGGTT
	ORF3a-A-LF	TGAAGAAGGTAACATGTTCAACACC
	ORF3a-A-LB	CGGAGTTGTTAATCCAGTAATGGAA
**LAMP ORF3a-B**	ORF3a-B-F3	ATTTTGTTCGCGCTACTG
	ORF3a-B-B3	ACGAGCAAAAGGTGTGAG
	ORF3a-B-FIP	CTCTGAAAAACAGCAAGAAGTGCAA-GATACCGATACAAGCCTCAC
	ORF3a-B-BIP	TAACCCTCAAAAAGAGATGGCAA-CAAACAACAACAGCAAGTTG
	ORF3a-B-LF	ACAATAAGCCATCCGAAAGGGA
	ORF3a-B-LB	CACTCTCCAAGGGTGTTCACT
**T7-LAMP-Primers**	GENE-N-T7-FIP	TCTGGCCCAGTTCCTAGGTAGT TAATACGACTCACTATAGG CCAGACGAATTCGTGGTGG
	ORF7-T7-FIP	TCCAGAAGAGCAAGGTTCTTTTAAA TAATACGACTCACTATAGG GCTACTTGTGAGCTTTATCACT
	ORF3a-A-T7-FIP	GTTCTTCAGGCTCATCAACAATTTT TAATACGACTCACTATAGGG CTCAACTCAATTGAGTACAGACA
**LwaCas13a crRNA**	ORF1ab[2]	GAUUUAGACUACCCCAAAAACGAAGGGGACUAAAAC CCAACCUCUUCUGUAAUUUUUAAACUAU
	S Gene[2]	GAUUUAGACUACCCCAAAAACGAAGGGGACUAAAAC GCAGCACCAGCUGUCCAACCUGAAGAAG
	Gene-N	GAUUUAGACUACCCCAAAAACGAAGGGGACUAAAAC TTGTTAGCACCATAGGGAAGTCCAGCTT
	Orf7	GAUUUAGACUACCCCAAAAACGAAGGGGACUAAAAC GGAUGAAAUGGUGAAUUGCCCUCGUAUG
	Orf3	GAUUUAGACUACCCCAAAAACGAAGGGGACUAAAAC UGUGAAUUUGGACAUGUUCUUCAGGCUC
**RPA Primers**	Orf1ab-Fw_v1[2]	GAAATTAATACGACTCACTATAGGGCGAAGTTGTAGGAGACATTA TACTTAAACC
	Orf1ab-Rev_v1[2]	TAGTAAGACTAGAATTGTCTACATAAGCAGC
	S-RPA-Fw_v1[2]	GAAATTAATACGACTCACTATAGGGAGGTTTCAAACTTTACTTGCT TTACATAGA
	S-RPA-Rev_v1[2]	TCCTAGGTTGAAGATAACCCACATAATAAG
**Reporter SHERLOCK2**	LF Reporter[2]	/56-FAM/mArArUrGrGrCmAmArArUrGrGrCmA/3Bio/
	[1] Sequence from Zhang et al, medRxiv: 2020.02.26.20028373.	
	[2] Sequence from the webpage of Feng Zhang @ MIT, Boston, USA	
