# Supplementary material for: Rapid SARS-CoV-2 testing in primary material based on a novel multiplex RT-LAMP assay
Source: PLoS One. 2020 Nov 2;15(11):e0238612. doi: 10.1371/journal.pone.0238612 (PMC7605681; doi:10.1371/journal.pone.0238612)
Supplement: S2 Table — (PDF) [file pone.0238612.s006.pdf]

		SARS-CoV2 qPCR	
Characteristics	**all**	**(+)**	**(-)**
n, male (%)	171* (56.97)	125 (58.40)	46 (54.34)
Age groups (n)			
<30	38	27	11
30-45	52	37	15
45-60	46	42	4
>60	35	19	16
Age, (yr) mean ± SD (all)	44.60 ± 18.50	44.74 ± 16.34	44.22 ± 23.59
Age, (yr) mean ± SD (male)	43.37 ± 17.74	43.66 ± 16.07	42.53 ± 22.28
Age, (yr) mean ± SD (female)	46.25 ± 19.47	46.26 ± 16.76	46.23 ± 25.46
Chest CT (n)	18	11	7

** date of birth was not available for one female patient (who was consequently not included in this table).*
